# Weekly administration of docetaxel in combination with estramustine and celecoxib in patients with advanced hormone-refractory prostate cancer: final results from a phase II study

**DOI:** 10.1038/sj.bjc.6604030

**Published:** 2007-10-23

**Authors:** J Carles, A Font, B Mellado, M Domenech, E Gallardo, J L González-Larriba, G Catalan, J Alfaro, A Gonzalez del Alba, M Nogué, P Lianes, J M Tello

**Affiliations:** 1 Department of Medical Oncology, Hospital del Mar-URTEC, Barcelona, Spain; 2 Department of Medical Oncology, Hospital Universitari Germans Trias i Pujol, Barcelona, Spain; 3 Department of Medical Oncology, Hospital Clinic i Provincial, Barcelona, Spain; 4 Department of Medical Oncology, Centre Althaia, Barcelona, Spain; 5 Department of Medical Oncology, Centre Hospitalari Parc Taulí, Barcelona, Spain; 6 Department of Medical Oncology, Hospital Clinico San Carlos, Madrid, Spain; 7 Department of Medical Oncology, Hospital de Son Llatzer, Palma de Mallorca, Spain; 8 Department of Medical Oncology, Hospital de Terrasa, Barcelona, Spain; 9 Department of Medical Oncology, Hospital de Son Dureta, Palma de Mallorca, Spain; 10 Department of Medical Oncology, Hospital General de Vic, Barcelona, Spain; 11 Department of Medical Oncology, Hospital de Mataró, Barcelona, Spain; 12 Department of Medical Oncology, Instituto de Oncología Corachán, Barcelona, Spain

**Keywords:** COX-2 inhibitor, docetaxel, estramustine, celecoxib, prostate cancer, androgen-independent

## Abstract

The objective of this study was to evaluate the efficacy and safety profile of weekly docetaxel, estramustine and celecoxib in patients with advanced hormone-refractory prostate cancer. Forty-eight patients received 35 mg m^−2^ of weekly docetaxel for 3 out of every 4 weeks, 280 mg of estramustine twice daily on days 1–3, 8–10, 15–17 and 400 mg of celecoxib twice daily until progression or toxicity. Cycles were repeated every 28 days for at least six cycles. Patients were evaluated for response and toxicity. Patients received a median of four cycles (range: 1–9). On an intention-to-treat analysis, prostate-specific antigen (PSA) was decreased greater than 50% in 28 out of 48 patients (overall response rate: 58%, 95% confidence interval (CI): 44–72) and median duration of PSA response was 8.0 months (95% CI: 6.9–9.0). After a median follow-up of 11.3 months, the median time to progression was 7.1 months and the median overall survival was 19.2 months. The most frequent severe toxicity was asthenia (15% of patients), diarrhoea and stomatitis (8% of patients, each). Grade 3/4 neutropenia was reported in two patients. There was a toxic death during the study due to a gastric perforation. Celecoxib with weekly docetaxel and estramustine is an effective and safe treatment for patients with hormone-refractory prostate cancer, but it does not seem to add any benefit to docetaxel.

Prostate cancer is the most common cancer among men in developed countries. Every year, there are approximately 680 000 new cases diagnosed and 220 000 deaths around the world (Ferlay *et al*, 2002). Despite improvements in early detection, about 10–20% of men with prostate cancer present with metastatic disease, and 33% of men with early prostate cancer will develop metastases despite local curative therapies, such as surgery or radiotherapy. Based on past experience, the median time to metastases is about 8 years from the time of surgery in those men who have a prostate-specific antigen (PSA) recurrence post-prostatectomy (Pound *et al*, 1999). Treatment of advanced or metastatic prostate cancer is palliative. In about 80% of men, primary androgen ablation leads to symptomatic improvement and a reduction in serum levels of PSA, but eventually in most patients, the tumour becomes androgen-independent after 18–24 months of castration (Crawford *et al*, 1989). Later remissions with additional hormonal therapies are less frequent and for shorter periods of time. Patients with advanced hormone-refractory prostate cancer (HRPC) have a progressive morbid disease with a median survival of 10–12 months.

Until recently, chemotherapy was considered to be ineffective in HRPC (Yagoda and Petrylak, 1993). Mitoxantrone plus prednisone (MP) palliates bone pain in 30% of patients but does not increase survival (Tannock *et al*, 1996). Two recent randomised phase III trials have shown a statistically significant survival benefit with docetaxel in combination with estramustine (Petrylak *et al*, 2004) or prednisone (Tannock *et al*, 2004) when compared to the standard MP treatment. In a recent randomised phase II study, weekly docetaxel in combination with estramustine and prednisone (EP) yielded similar efficacy results to those obtained by the standard triweekly docetaxel plus EP, and both were significantly superior to MP in terms of time to progression and PSA response (Oudard *et al*, 2005). Unfortunately, the survival benefit found in the TAX327 later on was only detected with triweekly docetaxel, but not with the weekly schedule. Apart from that, TAX327 trial showed a different toxicity profile for weekly *vs* triweekly docetaxel, specially regarding the incidence of severe neutropenia that was more frequently observed in the triweekly rather than in the weekly schedule (32 *vs* 2%, respectively) (Tannock *et al*, 2004).

Celecoxib belongs to a family of drugs called nonsteroidal anti-inflammatory drugs (NSAIDs). It is a selective cyclooxygenase-2 (COX-2) inhibitor. Aberrant or increased expression of COX-2 has been implicated in the pathogenesis of many diseases including cancer. Cyclooxygenase-2 also has a key role in a wide variety of processes related to carcinogenesis, including inhibition of apoptosis, increased cell growth, increased cell migration, inhibition of the immune response and stimulation of angiogenesis (Gumgumji *et al*, 2003).

Selective COX-2 inhibitors may play an important role in the treatment of prostate cancer, because overexpression of COX-2 is observed in the majority of human prostate carcinomas (Gupta *et al*, 2000) and these agents have demonstrated the ability to suppress tumour growth in prostate cancer cell lines (Liu *et al*, 2000). However, there are safety concerns to be taken into consideration when selective COX-2 inhibitors are taken regularly for long periods of time as they have been found to be associated with an increased incidence of cardiovascular side effects.

To address these concerns, we carried out a prospective phase II study to evaluate the efficacy and safety profile of weekly docetaxel in combination with estramustine and celecoxib for the treatment of patients with HRPC. The primary objective of the study was to determine PSA response to study treatment. Secondary objectives included the assessment of clinical response, time to progression, overall survival and the safety profile.

## MATERIALS AND METHODS

### Study design

This was a multicenter, phase II, prospective, open label, non-randomised trial in 12 hospitals in Spain. Recruitment of patients took place from October 2003 to June 2005. The Institutional Review Board of each participating site approved the study protocol. All patients provided their written informed consent. Clinical Research Forms were specifically designed to record the study data and source data verification was performed on 100% of study information.

### Selection of patients

Eligibility criteria included histologically confirmed adenocarcinoma of the prostate and progressive metastatic disease in spite of androgen suppressive treatment. Progressive disease was defined as the occurrence of at least one of the following conditions: (1) increasing serum levels of PSA on two consecutive measurements obtained at least 1 week apart (with a minimum level of PSA >10 ng ml^−1^); (2) the appearance of new bone metastatic lesion(s) and (3) >20% increase in the size of a previously existing lesion. Patients had to be ⩾18 years old, with a Karnofsky Index ⩾60% and a life expectancy of longer than 3 months together with adequate bone marrow (neutrophils ⩾1.5 × 10^9^ l^−1^, platelets >100 × 10^9^ l^−1^ and haemoglobin ⩾10 g dl^−1^) and renal and hepatic function (serum creatinine <1.5 × upper normal limit (UNL); total bilirubin <1.25 × UNL, aspartate aminotransferase (ASAT) and alanine aminotransferase (ALAT) <2.0 × UNL). Antiandrogen therapy had to be discontinued for at least 4–8 weeks depending on the type administered. Patients continued taking luteinizing-hormone-releasing hormone agonists throughout the study treatment period in order to maintain androgen ablation. Prior radiotherapy and/or two previous hormonal therapies were permitted if at least 4 weeks had elapsed since the completion of those therapies.

Patients were excluded if they had been previously treated with chemotherapy. Other exclusion criteria were the presence of concomitant diseases including severe and uncontrolled cardiovascular disease, thromboembolic events in the last 6 months, peripheral neuropathy of grade 2 or higher, history of another cancer except those in complete remission for at least 2 years or other serious medical condition. Patients were also ineligible if they had hypersensitivity to celecoxib or allergy to estradiol, sulphonamides or NSAIDs.

This study was conducted in accordance with the standards of the Responsible Institutional Committees on Human Experimentation and the Helsinki Declaration of the World Medical Association amended in 1975 and subsequent revisions.

### Therapy

Eligible patients were treated with 280 mg of estramustine (Estracyt®, Pfizer Inc.) administered orally twice daily 1 h before, or 2 h after, meals on days 1–3, 8–10 and 15–17 of the treatment cycle. Celecoxib (Celebrex®, Pfizer Inc.) was administered at a dose of 400 mg orally twice daily from day 1 until progression of the disease or unacceptable toxicity. Docetaxel (Taxotere®, Sanofi-Aventis) 35 mg m^−2^ was administered as a 1-h intravenous infusion weekly on days 2, 9 and 16 of the treatment cycle. Treatment cycles were repeated every 28 days for at least 6 cycles. Study treatment could be prolonged until progression of the disease or unacceptable toxicity.

Prophylactic premedication included one dose of dexamethasone (8 mg) administered orally 1 h before each docetaxel infusion. Antiemetic and gastric protector agents were administered according to the usual practice at each hospital. No prophylactic anticoagulant therapy was administered in this study.

Blood counts and serum biochemistry tests were conducted before each cycle. If a patient showed a neutrophil count <1.5 × 10^9^ l^−1^ and/or a platelet count <100 × 10^9^ l^−1^, chemotherapy was delayed for 1 week. If toxicity persisted after 3 weeks, the patient was withdrawn from the study. Docetaxel dosage was reduced to 75% in the case of febrile neutropenia, prolonged neutropenia (neutrophils <0.5 × 10^9^ l^−1^ for longer than 5 days), grade 3/4 thrombopenia along with haemorrhage or ⩾2 weeks delay in treatment due to toxicity. Patients were withdrawn from the study after three or more reductions in docetaxel dosage. In addition, study treatment was delayed for 1 week in the case of grade 2 or higher non-haematological toxicity, excluding nausea and vomiting, for a maximum of 2 weeks. Celecoxib was interrupted in the case of bilirubin exceeding 3.0 × UNL or elevation of hepatic aminotransferases (ASAT and/or ALAT) to >2.5 × UNL. Celecoxib therapy was restarted only when normal values or grade 1 toxicity were achieved.

### Evaluation of safety and response

Pre-study evaluations included complete medical history, the recording of weight, height and Karnofsky performance status, electrocardiogram, haematology and biochemistry tests including measurement of serum PSA, and other examinations as clinically indicated. Tumour measurements were performed by imaging-based evaluations including computed tomography of the abdomen and pelvis, bone scanning and thorax radiography. Tumour measurements were assessed at baseline and after two, four and six treatment cycles.

Objective clinical response was evaluated according to the Response Evaluation Criteria in Solid Tumors (RECIST) guidelines (Therasse *et al*, 2000).

Serum PSA was measured after each treatment cycle and was evaluated following recommendations from the Prostate-Specific Antigen Working Group (Bubley *et al*, 1999). A PSA response was defined when a confirmed decrease of at least 50% from baseline was observed. Duration of the PSA response was defined as the time between the first decrease of at least 50% of PSA from baseline until an increase of 50% (to at least 5 ng ml^−1^) over the nadir.

### Statistical analysis

The primary objective of the study was to determine PSA response to study treatment. Secondary end points included analysis of clinical response in measurable disease, time to progression, overall survival and toxicity.

The sample size was calculated using the Simon method for phase II trials. Taking into account a PSA response rate of 35% achieved in previous studies with the standard schedule of MP, we anticipated a response rate in PSA level of 55% with the new study treatment. With a type I error of 5% and a study power of 80%, the estimated sample size for this study was 44 evaluable patients.

Safety analyses were performed on patients who received at least one treatment cycle (safety population). All toxicities recorded were documented and graded according to the Common Toxicity Criteria of the National Cancer Institute (Version 2).

Efficacy analyses were conducted on the intent-to-treat population (efficacy population). Overall response rate (ORR) was calculated with 95% confidence intervals (CIs). Time to progression was defined as the period of time from the start of the treatment to first evidence of clinical or biochemical progression or death. Survival was calculated from the date of first administration of treatment to the date of death by any cause. Time to progression and overall survival were analysed using the Kaplan–Meier method.

## RESULTS

Between October 2003 and June 2005, a total of 48 patients were enrolled. The patient characteristics at baseline are shown in Table 1. The median Karnofsky index was 80% and the median time from first diagnosis was 37 months (range: 7–174 months). Sixty percent of patients had stage IV disease at diagnosis. The majority of patients (88%) had bone metastases and 31% of patients had lymph nodes affected. Only 19 patients (40%) were considered to have measurable disease. The median number of disease lesions per patient was 3.5. Median PSA at baseline was 70 (range: 1–1193 ng ml^−1^).

### Treatment

A total of 207 cycles of estramustine, celecoxib and docetaxel were administered, with a median number of four cycles per patient (range: 1–9 cycles). Patients received 602 docetaxel infusions with a median of 12 and a range of 3–27 infusions. However, 19 infusions of docetaxel (3% over 621 scheduled) needed to be suspended due to non-treatment-related causes (8, 42%), non-haematological toxicity (8, 42%), haematological toxicity (1, 5%), and both haematological and non-hematological toxicities (2, 11%). Docetaxel dosage was reduced in 16 infusions (3% over 602 administered) of which nine were due to non-haematological toxicity; six were due to non-drug-related causes and one due to haematological toxicity.

The number of estramustine dosages was 600 out of 621 scheduled (97%). Only four dosages of estramustine needed to be reduced due to non-haematological toxicity. The median absolute and relative dose intensity for docetaxel and estramustine was 25.1 mg m^−2^ week^−1^ (96%) and 178.8 mg day^−1^ (99%), respectively. Celecoxib was reduced in two patients, one due to patient decision and the other one because of grade 3 epigastric toxicity.

Twenty-three patients (48%) completed treatment according to protocol and 25 (52%) discontinued due to adverse events (*n*=13), patient decision (*n*=5), investigator criteria (*n*=3), death (*n*=3) and protocol violation (*n*=1).

### Efficacy

On an intention-to-treat analysis, PSA was decreased >50% in 28 out of 48 patients (ORR: 58%, 95% CI: 44–72). The median duration of PSA response was 8.0 months (95% CI: 6.9–9.0). Of 19 patients with measurable disease, one CR and six PR were observed (37%, CI 95%: 15–59), but only four of these were subsequentially confirmed. Moreover, nine patients (47%) showed SD and three patients (16%) progressed during the administration of study treatment.

**Figure 1:**
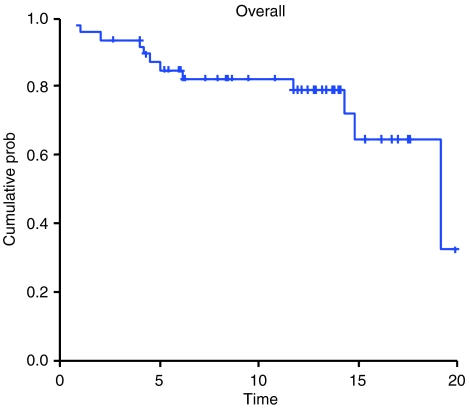
Overall survival (*n*=48).

After a median follow-up of 11.3 months, the median time to progression was 7.1 months (CI 95%: 5.3–8.8), and the median overall survival was 19.2 months (CI 95%: 12.9–25.4) (Figure 1). The median time to progression by PSA was 8.7 months (CI 95%: 7.7–9.8).

### Safety

All patients were evaluated for toxicity (Table 2). The most frequent grade 3/4 toxicity was asthenia (15% of patients, 4% of cycles). Other severe non-haematological toxicities were diarrhoea and stomatitis, each in of 8% of patients and 2% of cycles. Grade 3/4 anorexia, nausea and vomiting were reported in three patients (6%), one cycle each. Regarding severe haematological toxicities, anaemia was reported in three patients (6%) and six cycles (3%), and neutropenia was reported in two patients (4%), one cycle each (1%). The only severe cardiovascular events observed during the study were two patients with oedema (4%), one patient with a deep venous thromboembolism (2%) and one patient with a vasovagal syncope (*n*=1).

During the study, 13 patients (27%) were withdrawn from the study due to adverse events and three patients died (6%). Toxicity-related withdrawals were severe nail disorders (*n*=3), dyspnoea (*n*=2), cutaneous toxicity (*n*=2), asthenia (*n*=1), haematological toxicity (*n*=1), oedema (*n*=1), pain and poor general well-being (*n*=1), pulmonary embolism (*n*=1) and femur fracture (*n*=1). Reasons for patient death during study treatment administration were respiratory insufficiency (*n*=1), encephalopathy and renal failure (*n*=1) and there was one toxic death (*n*=1). The only death that could be possibly related to treatment was the toxic death. He was a 69-year-old man who, after the first cycle of treatment, suffered grade 1/2 diarrhoea and vomiting along with a severe antral gastric ulcer. The patient died 1 month later from a gastric perforation possibly associated with the use of celecoxib. The patient who died due to a respiratory insufficiency suffered a deterioration of a previous concomitant disease; and the encephalopathy and renal failure was supposed to be due to a locoregional progression.

## DISCUSSION

At this point in time, docetaxel is the only cytotoxic agent that has shown a significant improvement in overall survival, palliation of symptoms and improvement in quality of life for patients with HRPC (Mike *et al*, 2006) when compared with the standard combination of MP. The median overall survival achieved with docetaxel-based chemotherapy, administered with estramustine or prednisone, was close to 18 months (Petrylak *et al*, 2004; Tannock *et al*, 2004) and PSA declined at least 50% in ⩾45% of patients.

As far as we know, this study is the first reported phase II trial that has addressed the question of whether the addition of a selective COX-2 inhibitor such as celecoxib to this new standard chemotherapy regimen gives further clinical benefit in HRPC patients. Patients included in the study received an average of 25.1 mg m^−2^ week^−1^ of docetaxel and 178.8 mg day^−1^ of estramustine over 4 months. Celecoxib was administered at 800 mg day^−1^ from the beginning of the study until disease progression or until adverse events stopped the study. Efficacy results after this schedule showed a PSA response in 58% of patients and a median overall survival of 19.2 months, which are similar values to those found in previous studies where docetaxel/estramustine or docetaxel/prednisone was administered (Petrylak *et al*, 2004; Tannock *et al*, 2004). Oudard *et al* (2005) observed a median overall survival of 18.4 months and a PSA response rate of 63% when docetaxel was administered in a similar dosage and schedule (23.3 mg m^−2^ week^−1^) without celecoxib, but both estramustine and prednisone were administered concomitantly with docetaxel. It is known that the PSA response rate from prednisone alone is in the range of 16–24% (Tannock *et al*, 1996; Berry *et al*, 2002), and this fact could explain the results from that study and mask the activity of celecoxib in this indirect comparison.

The clinical benefit observed in our study with the addition of celecoxib to docetaxel/estramustine could have been even higher if docetaxel had been administered every 3 weeks instead of every week as recently reported (Tannock *et al*, 2004). However, only preliminary data from a randomised phase II trial in which weekly docetaxel plus EP yielded similar efficacy results to those obtained by standard triweekly docetaxel plus EP were available at the time of study design, and both were significantly superior than MP (Oudard *et al*, 2005).

Our results are in agreement with recently published studies where some evidence of the biological activity of celecoxib in patients with prostate cancer was observed (Pruthi *et al*, 2006; Smith *et al*, 2006). In a placebo-controlled study, celecoxib significantly decreased mean PSA velocity (*P*=0.02) and tended to increase the proportion of men who doubled their PSA-doubling time (Smith *et al*, 2006). In a second study, a slowing effect on the rate of PSA after 3 months of treatment with celecoxib was observed. This effect continued after 6, 12 and 18 months of treatment, suggesting that celecoxib may have an effect on serum PSA levels in patients with biochemical progression and therefore could delay or prevent disease progression (Pruthi *et al*, 2006).

One of the reasons for administering docetaxel on a weekly basis in our study instead of every 3 weeks was to avoid the high incidence of grade 3/4 neutropenia and the moderate incidence of thromboembolic events observed (Savarese *et al*, 2001). Certainly, the toxicity profile of our study treatment was tolerable. Grade 3/4 neutropenia was observed in only two patients (4% of patients), which compares well with the grade 3/4 neutropenia observed with docetaxel in a triweekly treatment schedule in other studies, such as CALGB 9780 (56% of patients) (Savarese *et al*, 2001), SWOG 9916 (16% of patients) (Petrylak *et al*, 2004) or TAX 327 trial (32% of patients) (Tannock *et al*, 2004). But it was very similar to that observed in the docetaxel weekly arm of TAX 327 trial (2% of patients). The incidence of other severe haematological and non-haematological toxicities was low. The most frequent grade 3/4 adverse events was asthenia (15% of patients), which is known to be one of the dose-limiting toxicities of weekly docetaxel (Hainsworth *et al*, 1998; Sitka Copur *et al*, 2001).

It has been reported that COX-2 inhibitors can minimise certain typical side effects of chemotherapy such as mucositis, diarrhoea and other inflammatory toxic effects. Cyclooxygenase-2 inhibitors may also be useful to control chronic pain that is very frequent in patients with advanced prostate cancer (Gasparini *et al*, 2003). However, during treatment administration, there was one toxic death. This was a 69-year-old man who, after the first cycle of treatment, suffered a severe antral gastric ulcer and gastric perforation that may have been associated with the use of celecoxib. It has been reported that celecoxib can delay healing in patients with complicated gastric ulcers, but at this point, only one previous case of gastric perforation has been reported to be associated with the use of celecoxib (Reuben and Steinberg, 1999).

In conclusion, this study shows that celecoxib in combination with weekly docetaxel plus estramustine is an effective and feasible treatment for patients with HRPC. The results obtained in this clinical trial may indicate that celecoxib could further improve the efficacy results obtained by docetaxel-based chemotherapy regimens in this type of patients. However, more studies like the STAMPEDE trial are needed to confirm these initial findings. The STAMPEDE trial is designed to assess the safety and efficacy of docetaxel, celecoxib and zoledronic acid given in various combinations. We will see if a clinical benefit is obtained with these drugs, and also if this benefit outweighs the potential risks associated with the long-term administration of selective COX-2 inhibitors.

## Figures and Tables

**Table 1:** Patient characteristics at baseline (*n*=48)

**Characteristic**	***N***	**%**
*Age (years)*		
Median	67	
Range	48–78	

*Karnofsky index (%)* a		
⩾80	37	77
<80	10	21

*Prior treatments*		
Prostatectomy	16	33
Radiotherapy	26	54
Estramustine	3	6
Hormonal therapy	48	100

*No. of previous hormonal treatments*		
1	19	40
2	16	33
>2	13	27

*Tumor stage*		
II	11	23
III	8	17
IV	29	60

*Sites of disease* b		
Bone	42	88
Soft tissue	15	31
Lymph node	3	6
Liver	3	6
Lung		

*No. of disease lesions*		
1	11	23
2	9	19
3	4	8
⩾4	24	50

a One patient missed.

b Patients could be included in more than one category.

**Table 2:** Grade 3/4 treatment-related toxicity (*n*=48)^a^

**Toxicity**	**Per patient (*n*=48) *N* (%)**	**Per cycle (*n*=207) *N* (%)**
*Haematological*		
Anaemia	3 (6)	6 (3)
Neutropenia	2 (4)	2 (1)

*Non-haematological*		
Asthenia	7 (15)	9 (4)
Diarrhoea	4 (8)	5 (2)
Stomatitis	4 (8)	4 (2)
Anorexia	3 (6)	3 (1)
Nausea	3 (6)	3 (1)
Vomiting	3 (6)	3 (1)
Dyspnea	2 (4)	3 (1)
Edema	2 (4)	2 (1)
Onycholisis	2 (4)	2 (1)

a Reported in two or more patients (⩾4% of patients).
